# Biomarker Dynamics and Long-Term Treatment Outcomes in Breast Cancer Patients with Residual Cancer Burden after Neoadjuvant Therapy

**DOI:** 10.3390/diagnostics12071740

**Published:** 2022-07-18

**Authors:** Milos Holanek, Iveta Selingerova, Pavel Fabian, Oldrich Coufal, Ondrej Zapletal, Katarina Petrakova, Tomas Kazda, Roman Hrstka, Alexandr Poprach, Maria Zvarikova, Ondrej Bilek, Marek Svoboda

**Affiliations:** 1 Department of Comprehensive Cancer Care, Masaryk Memorial Cancer Institute, Zluty kopec 7, 656 53 Brno, Czech Republic; holanek@mou.cz (M.H.); petrakova@mou.cz (K.P.); poprach@mou.cz (A.P.); zvarikova@mou.cz (M.Z.); bilek@mou.cz (O.B.); msvoboda@mou.cz (M.S.); 2 Department of Comprehensive Cancer Care, Faculty of Medicine, Masaryk University, Kamenice 753/5, 625 00 Brno, Czech Republic; 3 Research Centre for Applied Molecular Oncology, Masaryk Memorial Cancer Institute, Zluty kopec 7, 656 53 Brno, Czech Republic; hrstka@mou.cz; 4 Department of Pharmacology, Faculty of Medicine, Masaryk University, Kamenice 753/5, 625 00 Brno, Czech Republic; 5 Department of Oncological Pathology, Masaryk Memorial Cancer Institute, Zluty kopec 7, 656 53 Brno, Czech Republic; fabian@mou.cz; 6 Department of Breast, Skin and Oncoplastic Surgery, Department of Surgical Oncology, Masaryk Memorial Cancer Institute, Zluty kopec 7, 656 53 Brno, Czech Republic; coufal@mou.cz (O.C.); ondrej.zapletal@mou.cz (O.Z.); 7 Department of Surgical Oncology, Faculty of Medicine, Masaryk University, Kamenice 753/5, 625 00 Brno, Czech Republic; 8 Department of Radiation Oncology, Masaryk Memorial Cancer Institute, Zluty kopec 7, 656 53 Brno, Czech Republic; tomas.kazda@mou.cz; 9 Department of Radiation Oncology, Faculty of Medicine, Masaryk University, Kamenice 753/5, 625 00 Brno, Czech Republic

**Keywords:** breast cancer, neoadjuvant therapy, pathological complete response, residual cancer burden, biomarkers, KI-67, long-term outcomes

## Abstract

A residual cancer burden after neoadjuvant therapy (NAT) for breast cancer (BC) is associated with worse treatment outcomes compared to patients who achieved pathologic complete remission. This single-institutional retrospective study of 767 consecutive patients, including 468 patients with assessable residual cancer burden (aRCB) after NAT, with a median follow-up of 36 months, evaluated the biomarkers assessed before NAT from a biopsy and after NAT from a surgical specimen, their dynamics, and effect on long-term outcomes in specific breast cancer subtypes. The leading focus was on proliferation index Ki-67, which was significantly altered by NAT in all BC subtypes (*p* < 0.001 for HER2 positive and luminal A/B HER2 negative and *p* = 0.001 for TNBC). Multivariable analysis showed pre-NAT and post-NAT Ki-67 as independent predictors of survival outcomes for luminal A/B HER2 negative subtype. For TNBC, post-NAT Ki-67 was significant alone, and, for HER2 positive, the only borderline association of pre-NAT Ki-67 was observed in relation to the overall survival. Steroid and HER2 receptors were re-assessed just in a portion of the patients with aRCB. The concordance of both assessments was 92.9% for ER status, 80.1% for PR, and 92.2% for HER2. In conclusion, these real-world data of a consecutive cohort confirmed the importance of biomarkers assessment in patients with aRCB, and the need to consider specific BC subtypes when interpreting their influence on prognosis.

## 1. Introduction

Breast cancer (BC) is one of the most common malignancies, with an ever-increasing incidence, and one of the most common causes of death from cancer [1]. The goal of the treatment of early BC is to cure the disease, and all the available modalities of modern oncological systemic treatment are used for this purpose [2,3,4].

Neoadjuvant therapy (NAT) administered before definitive surgery is a standard treatment option in patients with BC, especially in aggressive subtypes, such as triple-negative breast cancer (TNBC) and human epidermal growth factor receptor 2 (HER2) positive breast cancer [2,4]. NAT pursues several goals, including breast-conserving surgery, axillary lymph node dissection omitted in patients with initially involved lymph nodes, and achieving pathological complete remission (pCR), which is defined as the complete disappearance of invasive cancer from the breast and axillary lymph nodes according to the definitive pathology examination of the tissue obtained by surgery [5]. Achieving pCR is associated with favorable long-term outcomes and a lower risk of recurrence and death [6,7,8,9], most pronounced in patients with TNBC and non-luminal HER2 positive BC [6,8].

A residual cancer burden (RCB) after NAT increases the risk of disease relapse and worsens overall survival (OS) [10,11,12]. For this reason, the aim is to provide the most effective therapy in the neoadjuvant setting, such as dose-dense chemotherapy regimens [13,14,15], dual anti-HER2 therapy in HER2 positive BC [16,17], platinum salts added in selected patients in TNBC [18,19,20,21,22], and, soon, administration of an immunotherapy and chemotherapy combination, which increases the likelihood of achieving pCR [23]. Despite the current possibilities of NAT, some patients have residual involvement in the breast or axillary lymph nodes after NAT due to several factors (e.g., different sensitivity to NAT in specific BC subtypes, treatment resistance, tumor biology, treatment toxicity, etc.). Even within this subgroup of patients with a high risk of disease relapse compared to patients who achieved pCR, the disease’s prognosis is different and associated with residual cancer burden characteristics [24] and biomarker changes [25].

The aim of this retrospective study on a single-institution consecutive cohort of BC patients neoadjuvantly treated outside clinical trials was to evaluate biomarkers assessed from the residual cancer burden in patients who did not achieve pCR and analyze their pre-NAT and post-NAT levels and their NAT-induced changes in relation to long-term treatment outcomes.

## 2. Materials and Methods

### 2.1. Patient Population and Follow-Up

**Figure 1:**
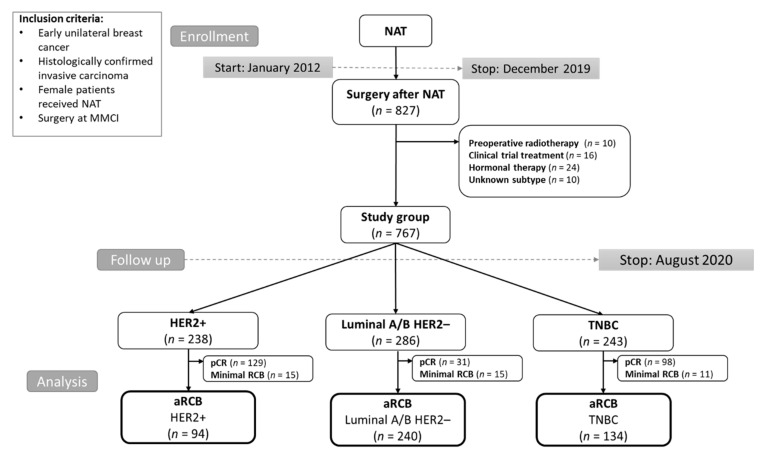
CONSORT diagram of the study population. Abbreviations: MMCI, Masaryk Memorial Cancer Institute; NAT, neoadjuvant therapy; TNBC, triple-negative breast cancer; HER2, human epidermal growth factor receptor 2; pCR, pathological complete response; aRCB, assessable residual cancer burden.

The study included female patients with histologically confirmed, nonmetastatic unilateral BC, who underwent surgery at Masaryk Memorial Cancer Institute between 2012 and 2019 and previously were treated with NAT. Patients with an unknown subtype, treated with neoadjuvant hormonal therapy, treated within clinical trials, and who underwent preoperative radiotherapy were excluded. The final analyzed group consisted of 767 consecutive patients (Figure 1). All patients provided written informed consent with the processing of their tissue samples for research purposes, and the study was allowed by the Institutional Ethics Committee of Masaryk Memorial Cancer Institute, protocol code 2019/3541/MOU, date of approval 17 December 2019. Patient follow-up during and after NAT was based on established standards of care in our institution and international guidelines. Follow-up included imaging and clinical examination; blood tests and additional imaging were performed in some cases (e.g., suspicion of disease relapse) based on the attending physician’s discretion. The follow-up schedule was as follows: in the first two years after 3–4 months, in the next three years every six months, and then once a year.

### 2.2. Pathological Assessment and Breast Cancer Subtype Classification

Histopathological records and results of core-cut biopsies and residual tissues after NAT were obtained from original pathological reports and reviewed. Tumor grade, expression of estrogen receptor (ER), progesterone receptor (PR), HER2 status, and Ki-67 proliferative index levels were assessed as follows: grading was determined by the Nottingham Histologic Score system in accordance with the WHO classification of breast tumors [26]; all immunohistochemical assays were performed using the Ventana Ultra Benchmark immunostainer (Roche Diagnostics, Basel, Switzerland). Monoclonal antibodies were used as follows: ER—SP 1 (Ventana/Roche); PR—NCL-PGR-312 (Novocastra/Leica Biosystems, Nussloch, Germany); Ki-67—30-9 (Ventana/Roche); HER2- 4B5 (Ventana/Roche). HER2 status was determined in accordance with the currently valid ASCO/CAP guidelines using immunohistochemistry or in situ hybridization [27]. According to the results of the pathological assessment from the core-cut biopsies, patients were divided into specific breast cancer subtypes (HER2 positive, luminal A/B HER2 negative, and TNBC). For purposes of this study, patients with negative or low expression (≤10%) for ER and PR and negative for HER2 were classified as TNBC [28]. Proliferative index Ki-67 was assessed in a 20× field with the highest proliferative activity (hot spot) by QuPath software [29]. According to the proliferative index Ki-67, patients were divided into four subgroups: the very low (≤10%), low to intermediate (11–40%), high (40–75%), and very high (>75%).

### 2.3. Residual Cancer Burden Assessment

Surgical specimen obtained after NAT was assessed by pathologists as a part of routine practice. pCR was defined as the complete disappearance of all invasive carcinoma from breast and axillary lymphatic nodes, while the presence of in situ carcinoma was allowed (ypT0/is ypN0) [5]. Patients with minimal RCB, such as pTmi or the presence of isolated tumor cells in lymph nodes, were excluded from further analysis due to the insufficient amount of the residual tumor tissue for biomarkers assessment and for the obtaining homogeneous cohort of patients with the assessable residual cancer burden (aRCB). The methods used to evaluate biomarkers in RCB were the same as for evaluating core-cut biopsies findings. Ki-67 expression was standardly assessed from surgical specimens if a sufficient amount of tissue was available. HER2 status was retested in patients with residual G3 tumors. Expression of ER, PR, and HER2 status was re-assessed in patients with heterogeneous residual tumors.

### 2.4. Long-Term Outcomes and Statistical Analysis

Long-term treatment outcomes were considered in terms of relapse-free survival (RFS) and overall survival (OS). RFS was determined as the time to the first event (locoregional relapse, distant relapse, or death from any cause), and OS was defined as the time to the date of death from any cause. Survival time was calculated from the date of surgery. Patients without the observed event or lost from follow-up were censored at the date of the last appropriate visit.

Patient and treatment characteristics were described using standard summary statistics, i.e., median and interquartile range (IQR) for continuous variables and frequencies and proportions for categorical variables. The logistic regression model was used to evaluate the association between Ki-67 and pCR. In patients for whom retesting steroid receptors expression was indicated, the agreement of steroid receptor status between biopsy and surgical specimens was evaluated using concordance analysis and Cohen’s kappa index. The Ki-67 levels between biopsy and surgical specimen were compared using Wilcoxon paired test. Survival probabilities were calculated using the Kaplan–Meier method [30]. Survival curves were compared using the log-rank test. The Cox proportional hazard model was used to perform the univariable and multivariable analysis and calculate hazard ratios (HR) [31]. The follow-up was determined using the reverse Kaplan–Meier method. All statistical analyses were performed employing R version 4.2.0 [32] and a significance level of 0.05.

## 3. Results

### 3.1. Baseline Characteristics and Treatment Response to NAT

The analyzed group included 767 retrospectively selected breast cancer patients treated by NAT. The median age at diagnosis was 49 years (range 17–85 years). The patient and tumor pretreatment characteristics and NAT regimens according to the BC subtypes are summarized in Table 1. The total pCR rate was 34%, specifically 54% for HER2 positive, 11% for luminal A/B HER2 negative, and 40% for TNBC. Achievement of pCR was not associated with pre-NAT Ki-67 for HER2 positive and TNBC patients (*p* = 0.260, *p* = 0.363, respectively). For luminal A/B HER2 negative patients, 31 patients achieving pCR had a significantly higher pre-NAT Ki-67 index (*p* < 0.001).

**Figure 2:**
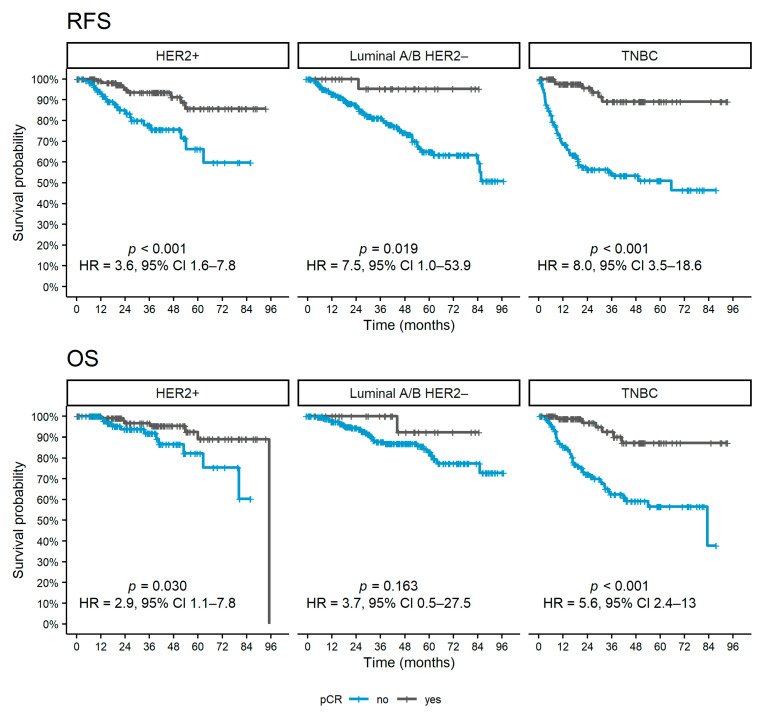
Kaplan–Meier curves of survival outcomes according to pCR achievement for each subtype. Abbreviations: pCR, pathological complete response; OS, overall survival; RFS, relapse-free survival; HR, hazard ratio; CI, confidence interval; TNBC, triple-negative breast cancer; HER2, human epidermal growth factor receptor 2.

During a median follow-up period after surgery of 36 months (95% CI 32–39), 153 (20%) relapses and 102 (13%) deaths were observed. The patients achieving pCR had a statistically significantly better RFS for all subtypes (*p* < 0.001 for TNBC and HER2 positive and *p* = 0.019 for luminal A/B HER2 negative, Figure 2) and OS for HER2 positive (*p* = 0.030) and TNBC patients (*p* < 0.001)

### 3.2. Residual Cancer Burden and Biomarker Dynamics after NAT

The patient cohort with aRCB had 468 patients (94 HER2 positive, 240 luminal A/B HER2 negative, 134 TNBC). The parameters assessed from the biopsy and surgical specimens for the aRBC group are summarized in Table 2.

Based on steroid receptors status assessed from the surgical specimen, concordance with the biopsy specimen was 92.9% (Cohen’s kappa 0.85) for ER status and 80.1% (Cohen’s kappa 0.59) for PR status regardless of BC subtype. The changes in steroid receptors status according to the BC subtypes are summarized in Table 3. A total of 10 (5%) HER2 negative patients from biopsy specimens changed status to HER2 positive on the surgical specimen after NAT; conversely, 10 (17%) patients shifted from HER2 positive to HER2 negative (concordance of 92.2%).

**Figure 3:**
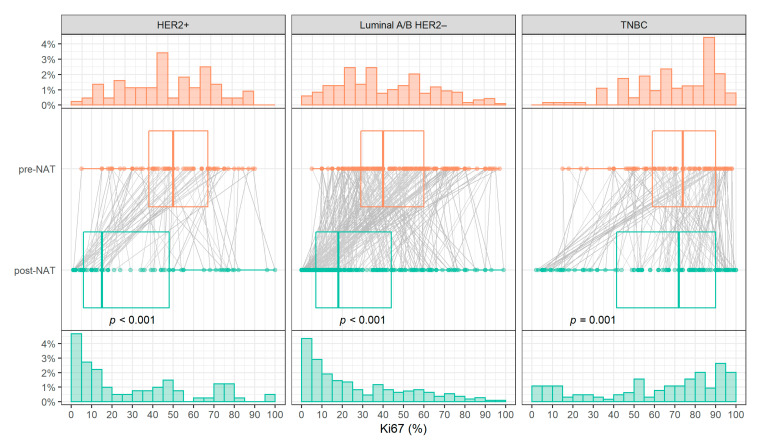
Histograms of Ki-67 pre- (top) and post-NAT (bottom) levels and comparison of Ki-67 levels (center). Abbreviations: TNBC, triple-negative breast cancer; HER2, human epidermal growth factor receptor 2; NAT, neoadjuvant treatment.

The Ki-67 expressions in the surgical specimens were highly significantly lower than in the biopsy for HER2 positive and luminal A/B HER2 negative (*p* < 0.001), while this decrease in Ki-67 expression was milder for TNBC patients (*p* = 0.001). Consequently, the Ki-67 levels distribution was shifted between biopsy and surgical specimens for HER positive and luminal A/B HER2 negative. Pre-NAT Ki-67 expressions were roughly uniformly distributed for these two subtypes. However, in post-NAT Ki-67 levels, the lower values predominated. In contrast, for TNBC, only a slight change in the level distribution was observed (Figure 3).

### 3.3. Ki-67 Expression and Long-Term Outcomes

**Figure 4:**
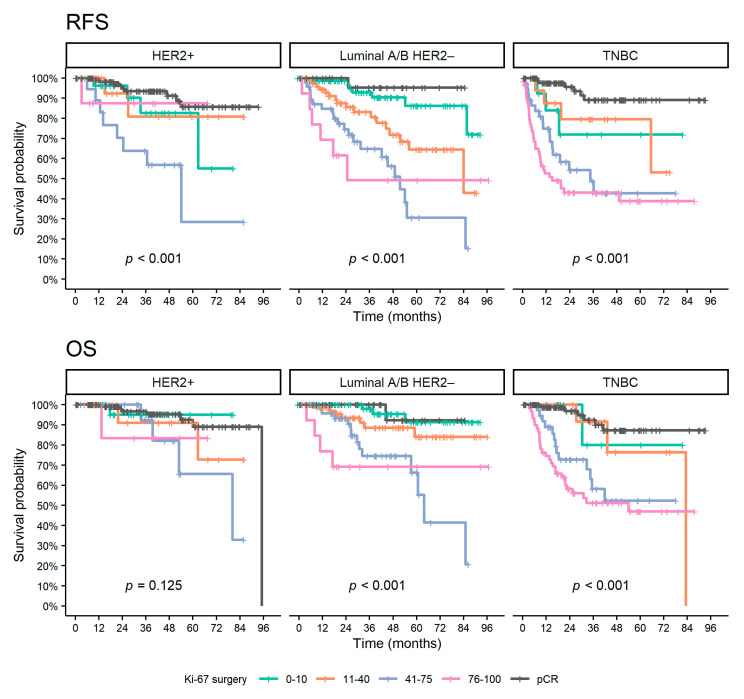
Kaplan–Meier curves of survival outcomes for patients with aRCB according to Ki-67 surgery and patients with pCR. Abbreviations: pCR, pathological complete response; OS, overall survival; RFS, relapse-free survival; HR, hazard ratio; CI, confidence interval; TNBC, triple-negative breast cancer; HER2, human epidermal growth factor receptor 2.

In the aRCB group, according to the subtype, the univariable analysis showed a prognostic effect of post-NAT Ki-67 levels and change in Ki-67 expressions between surgical and biopsy specimens for luminal A/B HER2 negative (HR = 1.24 and HR = 1.13 on continuous 10% scale, *p* < 0.001 and *p* = 0.009, respectively) and TNBC (HR = 1.14 and HR = 1.10 on continuous 10% scale, *p* = 0.005 and *p* = 0.044, respectively) patients. For luminal A/B HER2 negative patients, a statistically significant effect was also observed for pre-NAT Ki-67 (HR = 1.16 on a continuous 10% scale, *p* = 0.016). On the contrary, no significant association of Ki-67 levels with survival outcomes was noted for the HER2 positive subtype. The effect of post-NAT Ki-67 expression on long-term outcomes is shown in Figure 4. Luminal A/B HER2 negative patients with very low post-NAT Ki-67 levels showed favorable outcomes comparable to patients who achieved pCR. TNBC patients with very low to intermediate Ki-67 levels were represented in a smaller number, nevertheless with a higher risk of relapse than patients who achieved pCR, and a similar risk of death. Univariable analysis of other clinical and pathological pre- and post-NAT characteristics is summarized in Table 4 and Tables S1–S3.

According to multivariable analyses adjusted to age and pathological tumor and nodal stage (Table 5), pre-NAT and post-NAT Ki-67 were independently associated with RFS and OS for luminal A/B HER2 negative patients. On the other hand, RFS and OS were associated only with post-NAT Ki-67 for TNBC patients. For the HER2 positive subtype, the borderline significance of pre-NAT Ki-67 expression was observed in the multivariable analysis for RFS.

## 4. Discussion

This retrospective analysis of consecutive BC patients undergoing NAT focused on biomarkers commonly pathologically assessed for the biological characterization of breast tumors. The distinct biological BC subtypes respond differently to NAT, and, in addition, their prognostic and predictive factors are different [33,34,35,36]. The complete disappearance of invasive carcinoma from breast and axillary lymphatic nodes is associated with superior survival outcomes [6,7]; thus, it is one of the main goals of NAT. Our results, fully consistent with previously published studies [6,7], described a lower pCR rate for luminal A/B HER2 negative BC subtype. However, pCR achievement in these patients affected survival less. In aggressive breast cancer subtypes, such as TNBC and HER2 positive, the patients who did not achieve pCR were significantly more likely to relapse or die, as discussed elsewhere [10,37]. One of the known predictors of response to NAT is marker Ki-67 [34], used as a measure of tumor cell proliferation [38]. In our study, significantly higher levels of Ki-67 were observed only in luminal A/B HER2 negative patients with pCR. In other BC subtypes, this association was not observed, apparently with regard to higher Ki-67 levels of patients (median 51% and 77% in HER2 positive and TNBC, respectively).

Patients with RCB after NAT have a worse prognosis [10,11,12]. Therefore, determining prognostic and predictive factors in this group plays a crucial role in post-surgery therapeutic decisions. The biological characteristics of the tumor can be affected by anticancer therapy, and the pathological assessment of biomarkers is appropriate to repeat from the surgical specimen. Biomarker changes have been studied by many authors but with different results [39], and their impact on prognosis is thus still unknown. Moreover, differences in biomarker levels between biopsy and surgical specimens could be, on the one hand, treatment-related but also caused by the biological behavior of BC or by the limitations of biopsy (representativeness of biopsied specimen). Our study observed therapy-induced changes in HER2 status from positive to negative in 17% of retested specimens. Coiro et al. [25] described a lower frequency of changes in HER2 status; however, our results are influenced by indication criteria for retesting. Negative conversion of steroid receptors was more likely observed for PR compared to ER, similar to other studies, and is often associated with a worse prognosis [40,41].

Proliferative index Ki-67 is generally considered both a predictive [35] and prognostic factor [37,42]. However, there are some limitations in reproducibility due to inconsistent methodology of Ki-67 assessment, poor standardization, questionable analytical validity, or tumor heterogeneity [38,43]. Difficulties in unambiguous interpretation of Ki-67 levels are also related to inconsistent cut-off values for categorization [44]. Based on the results of our study, concerning the distribution of Ki-67 levels is appropriate for their categorization and interpretation to take into account the BC subtypes and whether the pathological assessment was performed from the biopsy or surgical specimen.

As shown, the Ki-67 levels in RCB can be used to separate patients with better outcomes. While TNBC patients with low to intermediate Ki-67 expression in RCB had a high rate of disease recurrence, according to the results of our study, patients with luminal A/B HER2 negative subtype with very low levels had compared survival outcomes to patients who achieved pCR.

One of the key observations of our study was the different dynamics of KI-67 expression between the biopsy and surgical specimen among BC subtypes. To the best of our knowledge, the previously published studies evaluated the prognostic role of Ki-67 dynamics in terms of absolute or relative differences [45] concordance using categorical evaluation [46] or direction of change [47]. These approaches do not consider both the baseline levels of Ki-67 and their NAT-induced changes. Accordingly, it is desirable to consider pre-NAT, post-NAT, or both Ki-67 levels according to the specific BC subtype to evaluate further treatment options and prognosis. In TNBC patients with RCB, Ki-67 expression generally remained at the same level compared to biopsy, and, according to the multivariable analysis, post-NAT Ki-67 levels are preferred for prognosis assessment. On the other hand, Ki-67 expression was not significantly associated with long-term outcomes in HER2 positive patients, except for border significance of baseline Ki-67 levels for OS. The greatest importance of Ki-67 assessment was observed in luminal A/B HER2 negative subtype, in which the results of the multivariable analysis proved pre- and post-NAT Ki-67 expressions to be independent prognostic factors.

We are aware of the study’s limitations, mainly caused by its retrospective nature. Firstly, not all the patients with aRCB were assessed for all the considered biomarkers due to standard indications for their retesting in clinical practice. Further, in the multivariable analysis, Ki-67 was considered a continuous variable, which is not easily transferable to clinical practice. Ki-67 cut-offs and categories are also not clearly defined, and there are some issues in their reproducibility, as discussed above. It would be very useful to perform further analyses to find unambiguous cut-off values. One of the possible limitations of our study is the length of the period from which the consecutive group of patients was included because the methods of assessment of biomarkers could be updated. Finally, the patient number in pertinent cohorts was not so large, particularly in the HER2 positive subgroup with aRCB, which may affect the statistical analysis. On the other hand, the reported data reflect the real clinical experience and could be used in daily practice.

Despite the above limitations, this study was performed on a large consistent cohort of patients and single-institution experience. The results may be considered both in the decision-making process for optimal treatment options and in the prognostication of patients.

## 5. Conclusions

The evaluation of biomarkers’ dynamic in aRCB in association with long-term outcomes proved desirable. The most effective way to predict the disease prognosis in patients with RCB after NAT is to consider not only the biomarkers’ levels in residual tissue but also their baseline values, taking into account specific BC subtypes. Ki-67 expression after NAT provides relevant independent and additional information in patients who did not achieve pCR. This approach allows the identification of patients with a high risk of disease relapse and optimization of patient management after surgery.

## Figures and Tables

**Table 1:** Patient and tumor pretreatment characteristics and NAT regimens according to the subtypes.

	Study Group (*n* = 767)			aRCB Group (*n* = 468)		
Variables	HER2+ *n* = 238	Luminal A/B HER2– *n* = 286	TNBC *n* = 243	HER2+ *n* = 94	Luminal A/B HER2– *n* = 240	TNBC *n* = 134
**Age (years)**						
Median (IQR)	52 (41, 62)	48 (40, 60)	46 (37, 58)	53 (40, 63)	48 (39, 60)	49 (37, 60)
Range	24, 85	20, 78	17, 78	25, 85	20, 78	23, 78
**Menopausal** **status**						
Pre	113 (47%)	151 (53%)	132 (54%)	42 (45%)	125 (52%)	68 (51%)
Peri/post	125 (53%)	135 (47%)	111 (46%)	52 (55%)	115 (48%)	66 (49%)
***BRCA1/2***						
Not tested	142 (60%)	160 (56%)	55 (23%)	52 (55%)	136 (57%)	37 (28%)
Wild type	90 (38%)	86 (30%)	114 (47%)	40 (43%)	79 (33%)	65 (49%)
Mutated	6 (2.5%)	40 (14%)	74 (30%)	2 (2.1%)	25 (10%)	32 (24%)
**cT**						
is	2 (0.8%)	0 (0%)	0 (0%)	0 (0%)	0 (0%)	0 (0%)
1	26 (11%)	38 (13%)	26 (11%)	7 (7.4%)	29 (12%)	11 (8.2%)
2	125 (53%)	164 (57%)	157 (65%)	47 (50%)	134 (56%)	80 (60%)
3	27 (11%)	48 (17%)	37 (15%)	13 (14%)	44 (18%)	27 (20%)
4	36 (15%)	21 (7.3%)	12 (4.9%)	19 (20%)	20 (8.3%)	8 (6.0%)
4d	22 (9.2%)	15 (5.2%)	11 (4.5%)	8 (8.5%)	13 (5.4%)	8 (6.0%)
**cN**						
0	69 (29%)	82 (29%)	98 (40%)	27 (29%)	64 (27%)	55 (41%)
1	147 (62%)	172 (61%)	124 (51%)	58 (62%)	146 (61%)	67 (50%)
2	15 (6.3%)	22 (7.7%)	18 (7.4%)	6 (6.4%)	20 (8.4%)	10 (7.5%)
3	7 (2.9%)	8 (2.8%)	3 (1.2%)	3 (3.2%)	8 (3.4%)	2 (1.5%)
Unknown	0	2	0	0	2	0
**Histology**						
NST	228 (96%)	269 (94%)	233 (96%)	89 (95%)	225 (94%)	131 (98%)
Other #	10 (4.2%)	17 (5.9%)	10 (4.1%)	5 (5.3%)	15 (6.2%)	3 (2.2%)
**Grade**						
1	1 (0.4%)	10 (3.6%)	1 (0.4%)	1 (1.1%)	10 (4.3%)	1 (0.8%)
2	74 (32%)	116 (42%)	21 (8.9%)	33 (36%)	106 (45%)	11 (8.5%)
2–3	24 (10%)	25 (9.0%)	12 (5.1%)	11 (12%)	23 (9.9%)	7 (5.4%)
3	135 (58%)	127 (46%)	201 (86%)	47 (51%)	94 (40%)	111 (85%)
NS	4	8	8	2	7	4
NAT regimens *						
A	0 (0%)	20 (7.0%)	9 (3.7%)	0 (0%)	17 (7.1%)	7 (5.2%)
A→cDDP	0 (0%)	8 (2.8%)	21 (8.6%)	0 (0%)	5 (2.1%)	9 (6.7%)
A→T	229 (96%)	241 (84%)	160 (66%)	88 (94%)	207 (86%)	89 (66%)
A→T + CBDCA	0 (0%)	16 (5.6%)	53 (22%)	0 (0%)	10 (4.2%)	29 (22%)
T	9 (3.8%)	1 (0.3%)	0 (0%)	6 (6.4%)	1 (0.4%)	0 (0%)
**Dose dense**	2 (0.8%)	5 (1.7%)	41 (17%)	0 (0%)	3 (1.3%)	18 (13%)

* all patients with HER2 positive BC were treated with chemotherapy plus anti-HER2 therapy (trastuzumab; trastuzumab + pertuzumab). # Other histological types include invasive lobular carcinoma (*n* = 8), invasive metaplastic carcinoma (*n* = 7), invasive micropapillary carcinoma (*n* = 7), invasive mucinous carcinoma (*n* = 4), invasive neuroendocrine carcinoma (*n* = 2), and rare histological types (*n* = 9). Abbreviations: TNBC, triple-negative breast cancer; HER2, human epidermal growth factor receptor 2; aRCB, assessable residual cancer burden; NST, invasive carcinoma of no special type; NAT, neoadjuvant therapy; A, anthracyclines; cDDP, cisplatin; T, taxane; CBDCA, carboplatin; NS, not specified; *BRCA 1/2*, breast cancer gene 1/2.

**Table 2:** Pathological pre- and post-NAT characteristics.

Variable	HER2+ *n* = 94		Luminal A/B HER2– *n* = 240		TNBC *n* = 134	
	Biopsy Specimen	Surgical Specimen	Biopsy Specimen	Surgical Specimen	Biopsy Specimen	Surgical Specimen
**pT**						
0, is		3 (3.2%)		19 (7.9%)		6 (4.5%)
1mi, 1		73 (78%)		138 (58%)		75 (56%)
2–4		18 (19%)		82 (34%)		52 (39%)
NS		0		1		1
**pN**						
0, itc		49 (52%)		72 (30%)		75 (56%)
1mi, 1		35 (37%)		94 (40%)		34 (26%)
2–3		10 (11%)		71 (30%)		24 (18%)
NS		0		3		1
**HER2**						
Negative	0 (0%)	10 (17%)	240 (100%)	114 (95%)	134 (100%)	75 (95%)
Positive	94 (100%)	49 (83%)	0 (0%)	6 (5.0%)	0 (0%)	4 (5.1%)
Not tested		35		120		55
**SR**						
Negative	27 (29%)	14 (24%)	0 (0%)	12 (8.5%)	133 (100%)	71 (91%)
Positive	66 (71%)	45 (76%)	240 (100%)	130 (92%)	0 (0%)	7 (9.0%)
Not tested	1	35	0	98	1	56
**ER (%)**						
Median (IQR)	80 (5, 100)	90 (0, 100)	100 (90, 100)	100 (95, 100)	0 (0, 0)	0 (0, 0)
Range	0, 100	0, 100	0, 100	0, 100	0, 10	0, 50
0–10	29 (31%)	19 (30%)	10 (4.2%)	14 (9.9%)	134 (100%)	84 (93%)
>10	64 (69%)	45 (70%)	230 (96%)	128 (90%)	0 (0%)	6 (6.7%)
Not tested	1	30	0	98	0	44
**PR (%)**						
Median (IQR)	15 (0, 80)	0 (0, 45)	58 (14, 90)	20 (0, 80)	0 (0, 0)	0 (0, 0)
Range	0, 100	0, 100	0, 100	0, 100	0, 10	0, 15
0–10	46 (49%)	33 (67%)	59 (25%)	65 (49%)	133 (100%)	76 (99%)
>10	47 (51%)	16 (33%)	181 (75%)	67 (51%)	0 (0%)	1 (1.3%)
Not tested	1	45	0	108	1	57
**Ki-67 (%)**						
Median (IQR)	50 (35, 67)	18 (6, 48)	40 (30, 60)	18 (7, 44)	74 (60, 90)	72 (41, 90)
Range	5, 90	1, 100	5, 97	0, 99	15, 98	2, 100
0–10	1 (1.1%)	30 (37%)	7 (3.0%)	80 (36%)	0 (0%)	14 (11%)
11–40	27 (31%)	23 (28%)	114 (48%)	79 (36%)	11 (8.7%)	18 (14%)
41–75	52 (59%)	20 (25%)	94 (40%)	50 (23%)	54 (43%)	38 (29%)
76–100	8 (9.1%)	8 (9.9%)	22 (9.3%)	11 (5.0%)	62 (49%)	59 (46%)
NS	6	13	3	20	7	5
**LVI**						
No		80 (85%)		154 (66%)		101 (76%)
Yes		14 (15%)		80 (34%)		32 (24%)
NS		0		6		1

Abbreviations: TNBC, triple-negative breast cancer; HER2, human epidermal growth factor receptor 2; ER, estrogen receptor; PR, progesterone receptor; SR, steroid receptors; LVI, persisted lymphovascular invasion; IQR, interquartile range; itc, isolated tumor cells; is, carcinoma in situ; mi, micrometastasis; NS, not specified.

**Table 3:** Change in steroid receptor status between biopsy and surgical specimens and concordance analysis for all subtypes.

	ER (Biopsy/Surgery)				PR (Biopsy/Surgery)			
	≤10/≤10	>10/>10	≤10/>10	>10/≤10	≤10/≤10	>10/>10	≤10/>10	>10/≤10
**HER2+** (*n*)	17 (27%)	40 (63%)	4 (6%)	2 (3%)	24 (50%)	13 (27%)	2 (4%)	9 (19%)
Concordance	90.5%				77.1%			
Cohen’s kappa (95% CI)	0.78 (0.61, 0.95)				0.53 (0.29, 0.76)			
**Luminal A/B HER2—**(*n*)	6 (4%)	127 (89%)	1 (1%)	8 (6%)	33 (25%)	60 (45%)	7 (5%)	32 (24%)
Concordance	93.7%				70.5%			
Cohen’s kappa (95% CI)	0.54 (0.28, 0.8)				0.41 (0.26, 0.55)			
**TNBC** (*n*)	84 (93%)	0	6 (7%)	0	75 (99%)	0	1 (1%)	0
Concordance	93.3%				98.7%			
Cohen’s kappa (95% CI)	NS				NS			

Abbreviations: TNBC, triple-negative breast cancer; HER2, human epidermal growth factor receptor 2; ER, estrogen receptor; PR, progesterone receptor; CI, confidence interval; NS, not specified.

**Table 4:** Univariable analysis of the association of clinical and pathological pre- and post-NAT characteristics with RFS and OS according to BC subtypes.

		RFS						OS					
		HER2+		Luminal A/B HER2–		TNBC		HER2+		Luminal A/B HER2–		TNBC	
		HR	*p*-Value	HR	*p*-Value	HR	*p*-Value	HR	*p*-Value	HR	*p*-Value	HR	*p*-Value
**Age**	10-years	1.30	0.106	1.24	0.061	0.87	0.173	1.75	**0.016**	1.59	**0.002**	0.97	0.759
**Menopausal status**	Pre	REF	**0.025**	REF	0.133	REF	0.286	REF	**<0.001**	REF	**0.008**	REF	0.773
	Peri/post	3.22		1.51		0.75		NS		2.80		0.92	
**cT**	1–2	REF	0.391	REF	**0.035**	REF	0.107	REF	0.058	REF	0.086	REF	0.141
	3,4,4d	1.50		1.79		1.57		3.86		1.90		1.58	
**cN**	0	REF	0.633	REF	**0.001**	REF	**0.007**	REF	0.063	REF	**0.003**	REF	**0.039**
	1–3	1.28		3.08		2.21		5.10		4.62		2.03	
**pT**	0,is,1,1mi	REF	0.268	REF	**0.024**	REF	**<0.001**	REF	0.242	REF	0.056	REF	**0.002**
	2–4	0.47		1.89		2.77		0.34		2.05		2.68	
**pN**	0,itc	REF	0.076	REF	**<0.001**	REF	**<0.001**	REF	0.509	REF	0.063	REF	**<0.001**
	1mi,1	2.36		2.14		2.13		1.53		1.23		2.05	
	2–3			5.66		6.14				2.67		5.60	
**Ki-67 biopsy** **(%)**	10% *	1.08	0.531	1.16	**0.016**	1.05	0.466	1.37	0.076	1.24	**0.010**	1.03	0.708
	0–10	REF	0.378	REF	0.087	REF	0.385	REF	0.132	REF	0.156	REF	0.723
	11–40												
	41–75	1.62		1.89		0.71		3.83		1.66		0.71	
	76–100			1.46		1.06				2.82		0.91	
**Ki-67 surgery** **(%)**	10% *	1.07	0.418	1.24	**<0.001**	1.14	**0.005**	1.14	0.279	1.33	**<0.001**	1.17	**0.004**
	0–10	REF	0.134	REF	**<0.001**	REF	**0.003**	REF	0.382	REF	**<0.001**	REF	**0.004**
	11–40	0.66		2.83				1.88		2.54			
	41–75	2.37		6.32		2.78		3.65		8.05		3.86	
	76–100			5.45		3.70				10.9		5.08	
**Difference in Ki-67 #**	10% *	1.02	0.853	1.13	**0.009**	1.10	**0.044**	0.96	0.741	1.16	**0.024**	1.15	**0.017**
**LVI**	No	REF	0.906	REF	**0.003**	REF	**<0.001**	REF	0.966	REF	0.067	REF	**0.001**
	Yes	1.08		2.29		3.76		0.97		1.98		2.83	

* Continuous 10% scale. # Difference between post-NAT and pre-NAT Ki-67 levels. Abbreviations: TNBC, triple-negative breast cancer; HER2, human epidermal growth factor receptor 2; OS, overall survival; RFS, relapse-free survival; HR, hazard ratio; CI, confidence interval; REF, reference category; LVI, persisted lymphovascular invasion. Significant *p*-Values (*p* < 0.05) are highlighted in bold.

**Table 5:** Multivariable analysis of Ki-67 expression adjusted to age and pathological tumor and nodal stage for RFS and OS according to BC subtypes.

			RFS			OS	
		HR	95% CI	*p*-Value	HR	95% CI	*p*-Value
** HER2+**							
Ki-67 biopsy	10% *	1.12	0.83, 1.52	0.462	1.55	0.94, 2.58	0.067
Ki-67 surgery	10% *	1.05	0.87, 1.27	0.594	0.98	0.70, 1.37	0.914
** Luminal A/B HER2–**							
Ki-67 biopsy	10% *	1.27	1.09, 1.49	**0.002**	1.27	1.03, 1.57	**0.025**
Ki-67 surgery	10% *	1.30	1.17, 1.44	**<0.001**	1.34	1.16, 1.55	**<0.001**
** TNBC**							
Ki-67 biopsy	10% *	1.05	0.89, 1.24	0.585	0.95	0.78, 1.16	0.600
Ki-67 surgery	10% *	1.10	0.99, 1.23	0.066	1.16	1.01, 1.34	**0.030**

* Continuous 10% scale. Abbreviations: TNBC, triple-negative breast cancer; HER2, human epidermal growth factor receptor 2; OS, overall survival; RFS, relapse-free survival; HR, hazard ratio; CI, confidence interval. Significant *p*-Values (*p* < 0.05) are highlighted in bold.

## Data Availability

The data presented in this study are available on request from the corresponding author.

## Supplementary Materials

The following supporting information can be downloaded at: https://www.mdpi.com/article/10.3390/diagnostics12071740/s1, Table S1: Univariable analysis for HER2 positive subtype; Table S2: Univariable analysis for luminal A/B HER2 negative subtype; Table S3: Univariable analysis for TNBC subtype.
