# Supplementary material for: Biomarker Dynamics and Long-Term Treatment Outcomes in Breast Cancer Patients with Residual Cancer Burden after Neoadjuvant Therapy
Source: Diagnostics (Basel). 2022 Jul 18;12(7):1740. doi: 10.3390/diagnostics12071740 (PMC9318288; doi:10.3390/diagnostics12071740)
Supplement: Supplementary file 1 [file diagnostics-12-01740-s001.zip › diagnostics-1801548-supplementary.pdf]

Table S1. Univariable analysis of the association of clinical and pathological pre- and post-NAT characteristics with RFS and OS for HER2 positive subtype

		RFS			OS		
		HR	95% CI	p-value	HR	95% CI	p-value
**Age**	10-years	1.30	0.95, 1.79	0.106	1.75	1.08, 2.81	**0.016**
**Menopausal status**	Pre	REF	—	**0.025**	REF	—	**<0.001**
	Peri/post	3.22	1.06, 9.80		NS		
**cT**	1-2	REF	—	0.391	REF	—	0.058
	3,4,4d	1.50	0.59, 3.82		3.86	0.81, 18.3	
**cN**	0	REF	—	0.633	REF	—	0.063
	1-3	1.28	0.45, 3.66		5.10	0.63, 41.3	
**pT**	0,is,1,1mi	REF	—	0.268	REF	—	0.242
	2-4	0.47	0.11, 2.05		0.34	0.04, 2.72	
**pN**	0,itc	REF	—	0.076	REF	—	0.509
	1mi,1,2-3	2.36	0.88, 6.30		1.53	0.43, 5.46	
**Ki67 biopsy**	10%*	1.08	0.85, 1.36	0.531	1.37	0.95, 1.98	0.076
	0-40	REF	—	0.378	REF	—	0.132
	41-100	1.62	0.53, 4.94		3.83	0.48, 30.5	
**Ki67 surgery**	10%*	1.07	0.91, 1.27	0.418	1.14	0.90, 1.45	0.279
	0-10	REF	—	0.134	REF	—	0.382
	11-40	0.66	0.12, 3.61		1.88	0.17, 21.1	
	41-100	2.37	0.73, 7.72		3.65	0.42, 31.4	
**Difference in Ki67**#	10%*	1.02	0.85, 1.21	0.853	0.96	0.75, 1.23	0.741
**LVI**	No	REF	—	0.906	REF	—	0.966
	Yes	1.08	0.31, 3.79		0.97	0.19, 4.82	

* Continuous 10% scale

Difference between post-NAT and pre-NAT Ki-67 levels

Abbreviations: TNBC, Triple-Negative Breast Cancer; HER2, human epidermal growth factor receptor 2; OS, overall survival; RFS, relapse-free survival; HR, hazard ratio; CI, confidence interval; REF, reference category; LVI, persisted lymphovascular invasion

Table S2. Univariable analysis of the association of clinical and pathological pre- and post-NAT characteristics with RFS and OS for luminal A/B HER2 negative subtype

		RFS			OS		
		HR	95% CI	p-value	HR	95% CI	p-value
**Age**	10-years	1.24	0.99, 1.54	0.061	1.59	1.18, 2.15	**0.002**
**Menopausal status**	Pre	REF	—	0.133	REF	—	**0.008**
	Peri/post	1.51	0.88, 2.59		2.80	1.27, 6.18	
**cT**	1-2	REF	—	**0.035**	REF	—	0.086
	3,4,4d	1.79	1.05, 3.06		1.90	0.92, 3.96	
**cN**	0	REF	—	**0.001**	REF	—	**0.003**
	1-3	3.08	1.44, 6.59		4.62	1.38, 15.4	
**pT**	0,is,1,1mi	REF	—	**0.024**	REF	—	0.056
	2-4	1.89	1.10, 3.25		2.05	0.99, 4.26	
**pN**	0,itc	REF	—	**<0.001**	REF	—	0.063
	1mi,1	2.14	0.87, 5.28		1.23	0.42, 3.55	
	2-3	5.66	2.46, 13.0		2.67	1.03, 6.90	
**Ki67 biopsy**	10%*	1.16	1.03, 1.31	**0.016**	1.24	1.05, 1.46	**0.010**
	0-40	REF	—	0.087	REF	—	0.156
	41-75	1.89	1.07, 3.33		1.66	0.74, 3.71	
	76-100	1.46	0.55, 3.88		2.82	0.98, 8.12	
**Ki67 surgery**	10%*	1.24	1.13, 1.36	**<0.001**	1.33	1.18, 1.51	**<0.001**
	0-10	REF	—	**<0.001**	REF	—	**<0.001**
	11-40	2.83	1.17, 6.84		2.54	0.66, 9.83	
	41-75	6.32	2.68, 14.9		8.05	2.29, 28.3	
	76-100	5.45	1.71, 17.3		10.9	2.43, 49.1	
**Difference in Ki67#**	10%*	1.13	1.03, 1.25	**0.009**	1.16	1.02, 1.33	**0.024**
**LVI**	No	REF	—	**0.003**	REF	—	0.067
	Yes	2.29	1.33, 3.94		1.98	0.96, 4.11	

* Continuous 10% scale

Difference between post-NAT and pre-NAT Ki-67 levels

Abbreviations: TNBC, Triple-Negative Breast Cancer; HER2, human epidermal growth factor receptor 2; OS, overall survival; RFS, relapse-free survival; HR, hazard ratio; CI, confidence interval; REF, reference category; LVI, persisted lymphovascular invasion

Table S3. Univariable analysis of the association of clinical and pathological pre- and post-NAT characteristics with RFS and OS for TNBC subtype

		RFS			OS		
		HR	95% CI	p-value	HR	95% CI	p-value
**Age**	10-years	0.87	0.72, 1.06	0.173	0.97	0.78, 1.20	0.759
**Menopausal status**	Pre	REF	—	0.286	REF	—	0.773
	Peri/post	0.75	0.44, 1.28		0.92	0.50, 1.67	
**cT**	1-2	REF	—	0.107	REF	—	0.141
	3,4,4d	1.57	0.92, 2.69		1.58	0.87, 2.90	
**cN**	0	REF	—	**0.007**	REF	—	**0.039**
	1-3	2.21	1.20, 4.06		2.03	1.00, 4.13	
**pT**	0,is,1,1mi	REF	—	**<0.001**	REF	—	**0.002**
	2-4	2.77	1.61, 4.76		2.68	1.45, 4.97	
**pN**	0,itc	REF	—	**<0.001**	REF	—	**<0.001**
	1mi,1	2.13	1.09, 4.19		2.05	0.90, 4.65	
	2-3	6.14	3.26, 11.6		5.60	2.65, 11.8	
**Ki67 biopsy**	10%*	1.05	0.91, 1.22	0.466	1.03	0.88, 1.21	0.708
	0-40	REF	—	0.385	REF	—	0.723
	41-75	0.71	0.27, 1.91		0.71	0.23, 2.18	
	76-100	1.06	0.41, 2.75		0.91	0.31, 2.66	
**Ki67 surgery**	10%*	1.14	1.03, 1.26	**0.005**	1.17	1.04, 1.32	**0.004**
	0-40	REF	—	**0.003**	REF	—	**0.004**
	41-75	2.78	1.08, 7.11		3.86	1.09, 13.7	
	76-100	3.70	1.55, 8.86		5.08	1.54, 16.8	
**Difference in Ki67#**	10%*	1.10	1.00, 1.21	**0.044**	1.15	1.02, 1.30	**0.017**
**LVI**	No	REF	—	**<0.001**	REF	—	**0.001**
	Yes	3.76	2.19, 6.45		2.83	1.54, 5.21	

* Continuous 10% scale

Difference between post-NAT and pre-NAT Ki-67 levels

Abbreviations: TNBC, Triple-Negative Breast Cancer; HER2, human epidermal growth factor receptor 2; OS, overall survival; RFS, relapse-free survival; HR, hazard ratio; CI, confidence interval; REF, reference category; LVI, persisted lymphovascular invasion
